# A comprehensive comparison of fluorescence in situ hybridization and cytology for the detection of upper urinary tract urothelial carcinoma: A systematic review and meta-analysis

**DOI:** 10.1097/MD.0000000000013859

**Published:** 2018-12-28

**Authors:** Hongyu Jin, Tianhai Lin, Jianqi Hao, Shi Qiu, Hang Xu, Ruichao Yu, Sheng Sun, Peng Zhang, Zhenhua Liu, Lu Yang, Liangren Liu, Ping Han, Qiang Wei

**Affiliations:** a Department of Urology, Institute of Urology, West China Hospital, Sichuan University; b West China School of Medicine, Sichuan University, Chengdu, Sichuan Province, China; c Department of Pathophysiology and Molecular Pharmacology, Joslin Diabetes Center; d Massachusetts General Hospital Cancer Center, Harvard Medical School, Boston, MA.

**Keywords:** cytology, FISH, sensitivity, specificity, UUT-UC

## Abstract

**Objective:**

To compare the relative effectiveness of fluorescence in situ hybridization (FISH) and cytology in diagnosing upper urinary tract urothelial carcinoma (UUT-UC) and to evaluate the advantages and potential deficiencies of FISH analysis.

**Methods:**

We performed a complete systematic review based on studies from PubMed/Medline, Embase, Web of Science, Ovid, Web of Knowledge, and Cochrane Library. We identified 2031 patients with strict criteria in 14 individual studies between January 2005 to November 2017 in accordance to preferred reporting items for systematic reviews and meta-analysis (PRISMA) guidelines, we summarized the test performance using bivariate random effects models.

**Results:**

FISH was superior to cytology in terms of pooled sensitivities (84.0%, 95% confidence interval [CI] 74.4–90.5% vs 40.0%, 95% CI 33.6–46.7%). FISH and cytology were similar to each other in terms of pooled specificities, which were 89.5% (95% CI 85.3–92.6%) for FISH and 95.9% (95% CI 91.2–98.1%) for cytology.

**Conclusion:**

We confirm the superiority of FISH over cytology in terms of sensitivity and find similar diagnostic outcomes between them based on systematic analysis. Therefore, we demonstrate that FISH is extremely sensitive while still very reliable with a relatively low error rate for diagnosing UUT-UC.

## 1 Introduction

Upper urinary tract (UUT) urothelial carcinoma (UUT-UC) is characterized by its wide range of grades and stages as well as its high tendency toward progression and recurrence,^[1,2]^ which threatens public health and well-being despite having a lower occurrence rate than that of other tumors in the urinary system.^[3]^ Therefore, an efficient, accurate yet non-invasive early-diagnostic technique is needed.^[4]^ Current diagnostic methods can be generalized into three categories, namely, cytology, imaging techniques, and endoscopy; notably, cytological examination is the most convenient and most widely applied method.

The UUT-UC has to be differentiated with urinary tract trauma, infection, renal cell carcinoma, and renal metastasis, as they manifest similar symptoms at early stage, such as hematuria, flank pain, or hydronephrosis. Imaging techniques such as computed tomography urography and intravenous pyelography fail to detect small tumors,^[5]^ and cytological examination also becomes far less efficient in terms of both sensitivity and specificity when detecting low-grade UUT-UC.^[6–8]^ More importantly, cytological examination can be subjective at times and is controversial in circumstances such as infection and inflammation.^[9,10]^ The accurate diagnostic rate of cytology increases to 50% to 60% when implemented with ureteroscopy, although the procedure-related complications are often inevitable.^[7,11]^ Theoretically, ureteroscopy is regarded as one of the standard methods in the diagnosis of UUT-UC. However, severe accompanying complications including infection, perforation, and hemorrhage can sometimes be unavoidable. In addition, anatomic abnormalities and a history of urinary tract reconstruction may render the ureteroscopy difficult and risky.^[12,13]^

In the last decade, fluorescence in situ hybridization (FISH), which is based on genetic aberrations and is associated with reduced complications,^[14]^ has exhibited high sensitivity and specificity in detecting UUT malignancies. Genetic mutations can be identified in the early stages of cancer development and represent important targets for clinical detection during further malignant transformation.^[15]^ FISH is known for its ability to analyze multiple chromosomal aberrations in a certain number of cells.^[16]^ Specifically, FISH detects aneuploidy in the 3rd, 7th, 9th, and 17th chromosomes in exfoliated cells collected from voided urine samples.^[17,18]^ Owing to the drastically increasing diagnostic accuracy of low-grade UC, the FISH probe set serves as an excellent supplement to cytological examination.^[19]^

During the last decade, many clinical trials comparing the newly established FISH analysis method and traditional methods, particularly cytology, have been initiated.^[3,9]^ However, some of these studies have included insufficient number of patients, and the results reported in various articles differ greatly, leading to incomplete and inaccurate conclusions.^[3,15,18]^ Therefore, the aim of this meta-analysis is to integrate the main parameters (sensitivity and specificity) along with secondary parameters, including PPV and NPV, to generate more reliable and authentic data.

## 2 Results

### 2.1 Search results

After careful resources’ searching in authenticated databases, article screening, and quality assessment process, 14 studies with high reliability, adequate sample size, and comprehensible design with accessible data and full texts were considered for this systematic review. The total number of patients incorporated was 2031. All studies were carried out in a retrospective and single-centered fashion. In terms of the nationalities and regions, 4 studies were performed in Europe, 8 in Asia, and 2 in North America. To select proper patient groups, most articles included patients with symptoms such as hematuria, hydroureterosis, or hydronephrosis and patients with suspected or readily diagnosed UUT-UC. Meanwhile, patients without adequate cell numbers in voided urine samples as required by FISH or with an insufficient follow-up duration, or patients with concomitant bladder carcinoma were excluded.

### 2.2 Characteristics of the included studies

Supplementary Table 1 shows detailed characteristics of the 14 included articles. The sensitivity and specificity of both diagnostic techniques were carefully calculated according to the exact number of patients with true positive (TP), true negative (TN), false positive (FP), or false negative (FN) diagnostic results. The pooled sensitivity of FISH was 84.0%, ranging from 51.9% in a study with 80 participants to 100% in a study with 12 participants. The pooled sensitivity of cytology was 40.0%, varying from 20.8% to 60%, which was relatively lower than that of FISH. We also examined the distribution of diagnostic sensitivity and specificity percentages between FISH and cytology (Table 1). The sensitivity of FISH exceeded that of cytology in both exact percentage and distribution, whereas the specificities of both diagnostic techniques were comparable.

**Table 1:**
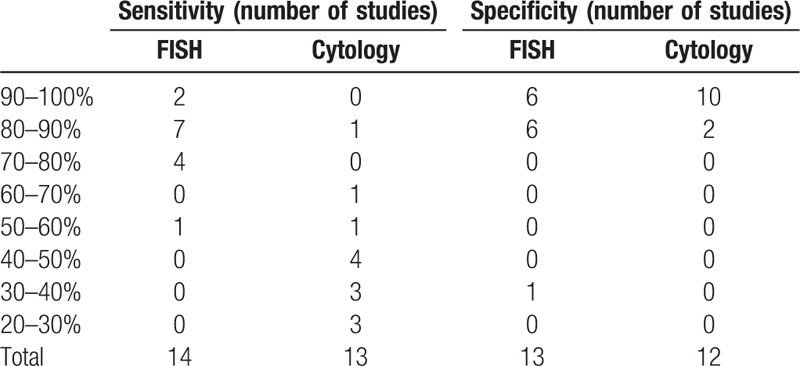
The distribution of diagnostic sensitivity and specificity percentages between fluorescence in situ hybridization (FISH) and cytology.

Several secondary parameters, including PPV and NPV, were also carefully extracted and evaluated (Table 2). Four of the 14 included studies also reported partial or complete alterations in chromosomes 3, 7, 9, and 17, as shown in Table 3.

**Table 2:**
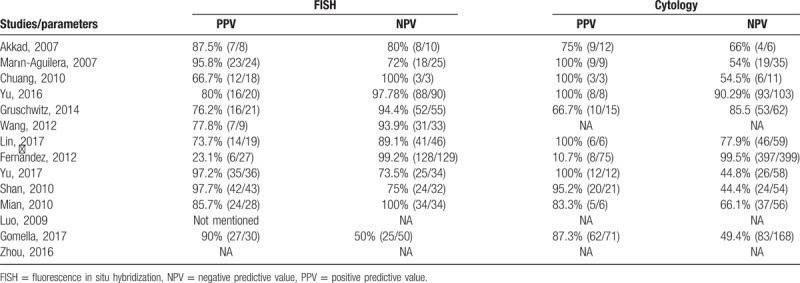
Comparison of PPV and NPV between FISH and cytology.

**Table 3:**
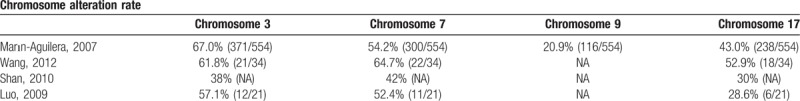
The alteration rate of some or all of chromosome 3, 7, 9, and 17.

### 2.3 Quality of the included studies

**Figure 1:**
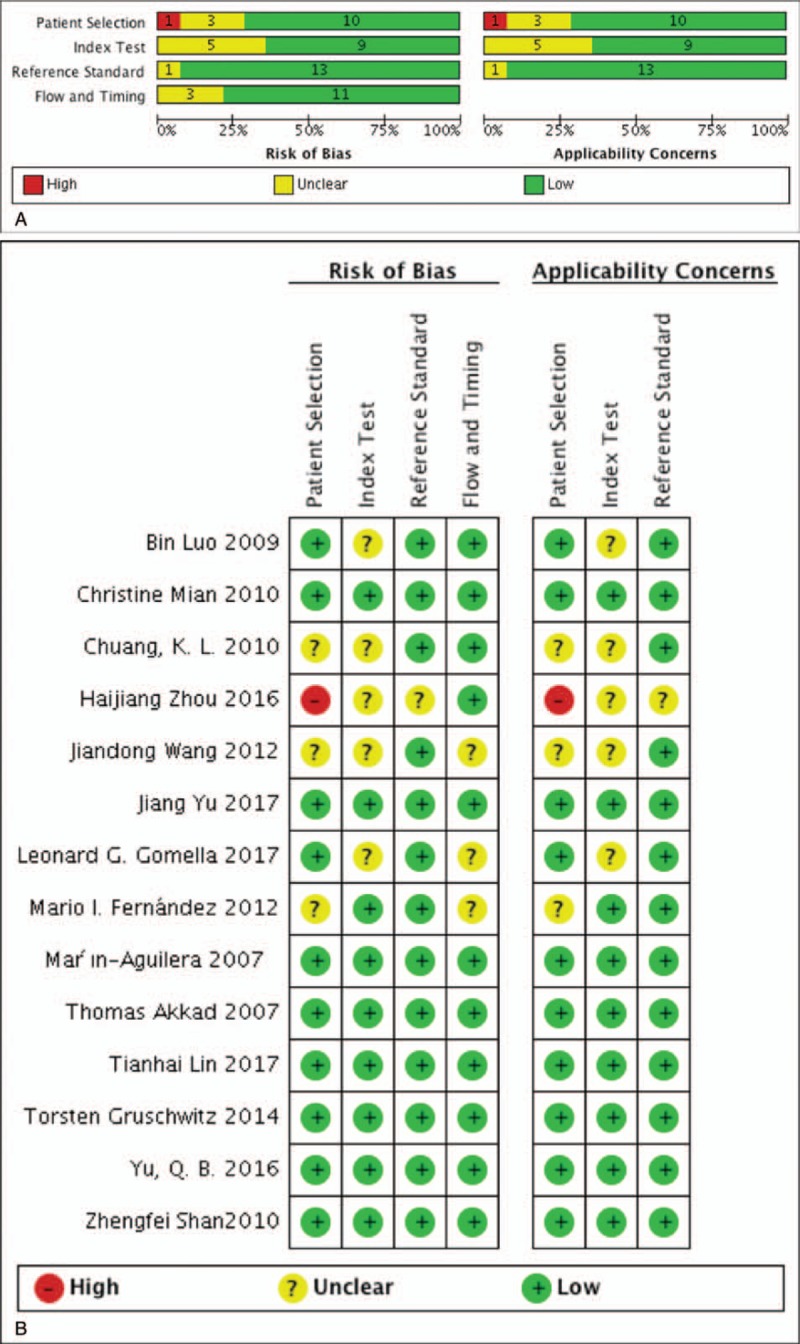
Quality assessment by Quadas-2 evaluation tool. (A) Risk of bias graph: review of authors’ judgments about each risk of bias item presented as percentages across all included studies. (B) Risk of bias summary: review of authors’ judgments about each risk of bias item for each included study.

Standard quality evaluation of the 14 included studies was performed based on the Newcastle–Ottawa scale^[20]^ (Supplementary Table 2), Quadas-2^[21,22]^ (Supplementary Table 3 and Fig. 1), and STARD 2015^[23]^ (Supplementary Table 4) tools. According to the three standard article evaluating systems, the 14 included studies were ultimately defined as reliable. However, some studies failed to fully describe the gold-standard comparator which may have led to low scores in index test. The method used to select patients may also have contributed to bias.

### 2.4 Parameters compared between cytology and FISH

**Figure 2:**
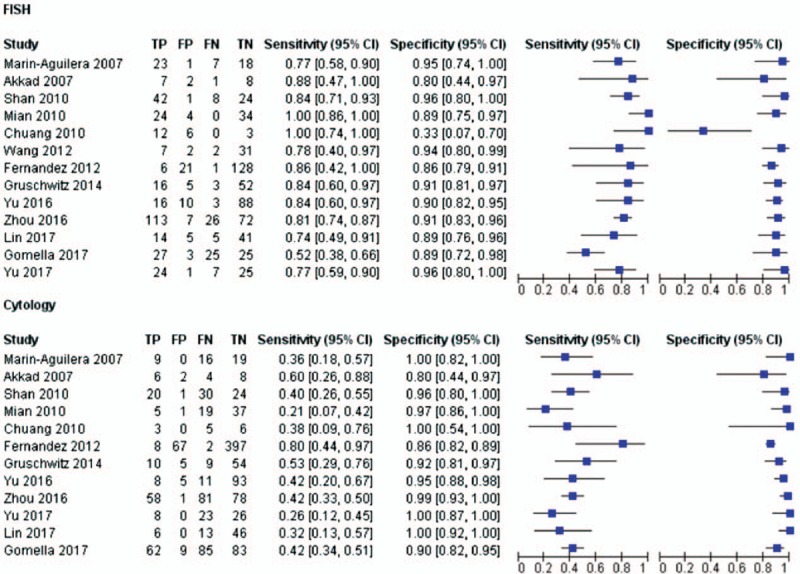
Forest plot comparing the sensitivity and specificity between fluorescence in situ hybridization and cytology.

To perform a comprehensive comparison regarding the efficiency and applicability of the 2 diagnostic techniques, we selected 12 key parameters for evaluation, including sensitivity, specificity, PPV, NPV, TP, TN, FP, and FN values, and the alteration rate for chromosomes 3, 7, 9, and 17 as mentioned above. A forest plot was, therefore, synthesized to manifest sensitivity, specificity, TP, TN, FP, and FN values of selected studies (13 studies for FISH and 12 studies for cytology) (Fig. 2), as one article lacks data on the specificity of both FISH and cytology, whereas another article doesn’t report sensitivity and specificity of cytology.

The pooled data showed no significant difference in specificity between FISH and cytology, which were 89.5% (95% CI 85.3–92.6%) for FISH and 95.9% (95% CI 91.2%-98.1%) for cytology, whereas the sensitivity of cytology was notably lower than that of FISH, which was 84.0% (95% CI 74.4%-90.5%) for FISH and 40.0% (95% CI 33.6%-46.7%) for cytology. The diagnostic ORs are 44.61 (95% CI 26.46–75.20%) and 15.47 (95% CI 7.68–31.17) for FISH and cytology, respectively. The LRs (+) and LRs (-) for FISH and cytology are 7.96 (95% CI 5.87–10.81%) vs 9.69 (95% CI 4.82–19.46%) and 17.9 (95% CI 11.1–28.9%) vs 62.6 (95% CI 56.9–69.0%), respectively.

## 3 Methods

### 3.1 Evidence acquisition

Authenticated databases including PubMed/Medline, Embase, Web of Science, Ovid, Web of Knowledge, and Cochrane Library were extensively searched for articles written in English published from January 2005 to November 2017 (full search strings available in the supplemental materials). We retrieved a total of 1112 articles. Thirty relevant studies remained after removal of obvious duplicates and meticulous correlational analysis. Of these, 5 studies were excluded because their full texts were inaccessible, and 7 articles were case reports, editorials, reviews, or letters. Finally, 14 studies were qualified for further analysis according to the following criteria: included more than 15 patients; reported sensitivity and specificity values for FISH and cytology; and randomized controlled trials and any observational design, including cross-sectional, case–control, and cohort designs.

**Figure 3:**
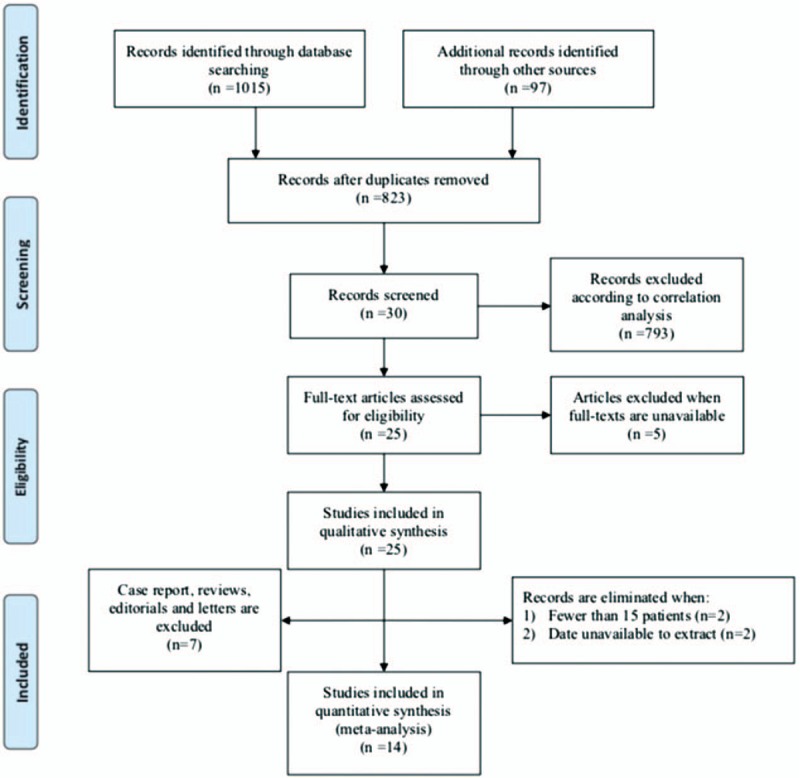
The flow diagram showing the article screening process.

Three independent reviewers participated in the screening process, analyzed the full texts, and performed quality assessments. Subsequently, we performed a blinded cross-check to detect underlying discrepancies. If a discrepancy was detected, a 4th reviewer was assigned to adjudicate the conflict. The identification, inclusion, and exclusion of studies were conducted according to preferred reporting items for systematic reviews and meta-analysis (PRISMA) guidelines. Figure 3 shows the PRISMA flow diagram of the article selection process.

### 3.2 Data extraction

A general data table containing 29 parameters was generated from the included articles by 3 individual reviewers simultaneously, and discrepancies were resolved through extensive discussions. The following information were extracted: title, author, nationality, department, ethnicity, study design, age and sex of the patients (both the experimental and control group), enrollment year, and comparison of correlated outcomes.

### 3.3 Statistical methods

Data were extracted on either an article or a study level when possible to reconstruct a 2 × 2 table, which was used to calculate sensitivity, specificity, PPV, NPV, ORs, and DLRs (Diagnostic Likelihood Ratio) along with the 95% CIs for each study. The forest plots were generated to display sensitivity and specificity estimates using Review Manager 5.3 (The Cochrane Collaboration). To summarize test performance, 2 methods for meta-analyzing diagnostic accuracy test have been used: the bivariate model^[24]^ and the hierarchical summary receiver operating characteristic (HSROC) model.^[25]^ We chose to use these methods to respect the binomial structure of diagnostic accuracy data, thus jointly summarizing paired measures simultaneously, for example, sensitivity and specificity or positive and negative likelihood ratios. Also, as a random effects approach, the bivariate/HSROC meta-analysis allowed pooling results in view of knowing that heterogeneity was commonplace across included studies due to different or implicit thresholds. The said approach was carried out by metandi (Meta-analysis of diagnostic accuracy using hierarchical logistic regression) command in STATA 14.2 (StataCorp).^[26]^

This study was approved by the Ethics Committee of West China Hospital, Sichuan University (Chengdu, China)

## 4 Discussion

This systematic review assessing the diagnostic accuracy of FISH and cytology for UUT-UC summarizes current literature and includes 14 studies for meta-analysis. The gathered evidence shows that FISH not only fits for an ideal alternative for diagnosing UUT-UC, but also proves much superior to cytology in terms of sensitivity. Recently, the most commonly applied diagnostic method for UUT-UC cytology has been reported to exhibit insufficient value in recognizing suspicious and ambiguous foci in the UUT,^[9,27,28]^ with the sensitivity as low as 30% to 50%.^[16,29]^ Regarding the potential harmful effect of ureteroscopy, a meta-analysis published in 2018 with 3975 patients indicated that diagnostic ureteroscopy had a negative impact on oncological outcomes, especially in intravesical recurrence,^[30]^ which was ascertained by another systematic review.^[31]^

As a novel biomolecular diagnostic technique, FISH has previously been reported to exhibit a relatively high sensitivity and specificity in detecting carcinoma in the urinary system, especially in the bladder.^[29]^ However, studies aimed to clarify the advancement and efficiency of FISH in the UUT were inadequately carried out.^[15,30,27]^ A study by Mian et al in 2010 with 68 patients demonstrated 100% sensitivity of FISH vs a considerably low sensitivity of 20.8% for cytology.^[32]^ In contrast, another study on 637 patients by Fernández et al in 2012 identified a much lower disparity in sensitivity between the 2 methods.^[28]^ Generally, the discrepancy between the sensitivities of FISH and cytology ranges from 5.7% to 89.2% for patient group sizes varying from 16 to 637.

Following a carefully considered and standardized process, we confirmed the higher sensitivity of FISH compared to that of cytology by standard statistical integration. In the specificity analysis, we obtained similar values between FISH and cytology, although the specificity of FISH was 6.4% lower than that of cytology. Conversely, Shan et al reported that an FP FISH result may imply potential tumor development in cases with a negative endoscopy or cytology result.^[4]^

Although FISH exhibits superior sensitivity and comparable specificity to those of cytology, inherent and inevitable challenges exist for FISH.^[9]^ So far, a number of opinions have been put forward regarding the potential deficiencies of FISH analysis method. First, compared with its excellent performance in diagnosing high-grade tumors, FISH is much less sensitive in detecting low-grade tumors.^[1]^ A likely explanation for more FN FISH results for low-grade tumors may be that tumor chromosomes are generally diploid or nearly diploid without obvious genetic abnormalities, which resemble those of normal cells.^[18]^ Second, the probe for 9p21, representing the most common site of genetic abnormalities, is the smallest in terms of size.^[33]^ Thus, non-typical, inconspicuous abnormalities are possible to be omitted at times. Finally, FISH requires collection of sufficient cell quantities in voided urine samples, which is difficult in some patients.^[34]^ Low cell quantities cannot provide the minimum number of chromosomes, thus precluding FISH analysis. Therefore, future optimization of cell collection either by washing urine or from voided urine rather than advances in cellular biochemical technologies alone is crucial.

In conclusion, this study pooled the largest number of UUT-UC patients to date and confirmed that FISH has higher sensitivity than cytology for diagnosing UUT-UC. However, we were unable to identify concrete differences in specificity between the 2 techniques. We acknowledge several limitations in this study. First, our study did not subgroup low- and high-grade tumors. Further studies may obtain distinct genetic aberration spectrums from different grades of tumor since they may have unique biological characteristics. Second, among the 14 eligible studies, only 4 contained detailed information regarding the alteration rates for chromosomes 3, 7, 9, and 17, and the exact numbers of chromosomal alterations evaluated varied substantially among studies. Therefore, the optimal cutoff values of specific chromosomal alterations remain to be determined.

## 5 Conclusion

We confirm the superiority of FISH over cytology in terms of sensitivity and found similar diagnostic outcomes between them based on systematic analysis. Therefore, we demonstrate that FISH is extremely sensitive while still very reliable with a relatively low error rate compared with cytology, which is valuable to clinical practice.

## Author contributions

**Conceptualization:** Hongyu Jin, Tianhai Lin, Qiang Wei.

**Data curation:** Hongyu Jin, Jianqi Hao.

**Formal analysis:** Hongyu Jin, Tianhai Lin.

**Funding acquisition:** Ping Han, Qiang Wei.

**Investigation:** Jianqi Hao, Shi Qiu.

**Methodology:** Lu Yang, Liangren Liu.

**Project administration:** Tianhai Lin, Qiang Wei.

**Resources:** Peng Zhang, Zhenhua Liu.

**Software:** Ruichao Yu, Hang Xu.

**Supervision:** Ping Han, Qiang Wei.

**Writing – original draft:** Hongyu Jin, Jianqi Hao.

**Writing – review & editing:** Tianhai Lin, Sheng Sun.
